# Characterizing the type 2 diabetes mellitus epidemic in Jordan up to 2050

**DOI:** 10.1038/s41598-020-77970-7

**Published:** 2020-12-03

**Authors:** Susanne F. Awad, Peijue Huangfu, Soha R. Dargham, Kamel Ajlouni, Anwar Batieha, Yousef S. Khader, Julia A. Critchley, Laith J. Abu-Raddad

**Affiliations:** 1 Infectious Disease Epidemiology Group, Weill Cornell Medicine–Qatar, Cornell University, Qatar Foundation - Education City, P.O. Box 24144, Doha, Qatar; 2 World Health Organization Collaborating Centre for Disease Epidemiology Analytics on HIV/AIDS, Sexually Transmitted Infections, and Viral Hepatitis, Weill Cornell Medicine –Qatar, Doha, Qatar; 3 Department of Population Health Sciences, Weill Cornell Medicine, Cornell University, New York, USA; 4 Population Health Research Institute, St George’s, University of London, London, UK; 5 The National Centre for Diabetes, Endocrine and Genetics, The University of Jordan, Amman, Jordan; 6 Department of Public Health and Community Medicine, Jordan University of Science and Technology, Irbid, Jordan

**Keywords:** Computational biology and bioinformatics, Applied mathematics, Statistics, Risk factors, Type 2 diabetes, Obesity

## Abstract

We aimed to characterize the type 2 diabetes mellitus (T2DM) epidemic and the role of key risk factors in Jordan between 1990–2050, and to forecast the T2DM-related costs. A recently-developed population-level T2DM mathematical model was adapted and applied to Jordan. The model was fitted to six population-based survey data collected between 1990 and 2017. T2DM prevalence was 14.0% in 1990, and projected to be 16.0% in 2020, and 20.6% in 2050. The total predicted number of T2DM cases were 218,326 (12,313 were new cases) in 1990, 702,326 (36,941 were new cases) in 2020, and 1.9 million (79,419 were new cases) in 2050. Out of Jordan’s total health expenditure, 19.0% in 1990, 21.1% in 2020, and 25.2% in 2050 was forecasted to be spent on T2DM. The proportion of T2DM incident cases attributed to obesity was 55.6% in 1990, 59.5% in 2020, and 62.6% in 2050. Meanwhile, the combined contribution of smoking and physical inactivity hovered around 5% between 1990 and 2050. Jordan’s T2DM epidemic is predicted to grow sizably in the next three decades, driven by population ageing and high and increasing obesity levels. The national strategy to prevent T2DM needs to be strengthened by focusing it on preventive interventions targeting T2DM and key risk factors.

## Introduction

The diabetes mellitus (DM) epidemic is a global public health concern and the seventh leading cause of death worldwide^1^. Besides demographic determinants^2^, it is established that the underlying epidemiology of the type 2 DM (T2DM) epidemic, which contributes greater than 90% of all DM cases^2^, is strongly influenced by modifiable risk factors such as obesity (defined as body mass index ≥ 30 kg/m^2^)^3–5^, smoking^6,7^, and physical inactivity^8,9^.

The Middle East and North Africa (MENA) region has the second highest DM prevalence worldwide, with 54.8 million adults (prevalence of 12.8%) estimated to be living with DM^2^. This number is projected to reach 107.6 million by 2045, an increase of 96%^2^. In Jordan, a MENA country and the focus of our study, several national population-based epidemiological surveys, conducted between 1994 and 2017, reported high but variable DM prevalence ranging between 9 and 30%^10–15^. Some also reported high and increasing prevalence of T2DM-related risk factors including obesity^10–15^, smoking^10–15^, and physical inactivity^15^.

Against this background, we aimed to project the temporal trends in prevalence and incidence of T2DM in Jordan up to 2050, factoring the interplay between T2DM natural history, T2DM-related risk factors, and demography. We further aimed to estimate the national heath expenditure directly attributed to T2DM, and to delineate the role of the modifiable risk factors of obesity, smoking, and physical inactivity in driving T2DM incidence and prevalence.

A highlight of this study is that it applied a recently-developed analytical approach to investigate T2DM epidemiology and its projections^16^. In contrast to earlier approaches^17–24^, this methodology captures the dynamic interactions between T2DM and its risk factors. Another highlight is that this study factored and synthesized the totality of existing evidence on T2DM and risk factors in Jordan to generate the different estimates—a total of six surveys for T2DM and its risk factors were used to calibrate the model.

## Methods

An age-structured T2DM mathematical model was developed to describe the natural history and progression of T2DM in the population of Jordan—as an adaptation of a published T2DM modelling approach^16^. Briefly, the natural history of T2DM was described by the general progression states of (1) four main susceptible classes: healthy (i.e. having none of the included T2DM-related risk factors), obese, smoker, and physically inactive; and (2) T2DM (including also all risk factor states). The susceptible and T2DM populations were further stratified to account for the overlaps between risk factors. The model disaggregated the population by sex and age to accommodate for the full demographic transition in the population. Accordingly, a total of 640 coupled differential equations were needed to describe T2DM epidemiology in this population. Further details on model structure and assumptions can be found in Awad et al.^16^ and in Supplementary Information Text S1.

### Data sources and model fitting

The parameters of the model were based on epidemiological and natural history data listed in Tables S2 and S3, and by fitting the model to existing prevalence and demographic measures.

The model was fitted to different survey data using a least-square fitting method^25^. This technique, implemented in MATLAB through the function FMINSEARCHBND^25^, minimizes the error function (i.e., cost function) between all data points and the model predictions starting from an initial set of parameter values, using the Nelder–Mead simplex algorithm as described in Lagarias et al.^26^ The Nelder–Mead algorithm is a commonly applied numerical method used to find the minimum of a function in a multidimensional space using the *simplex* method^26,27^. In our study, the sum of squared error was used as the cost function to provide a metric representing the difference between the data and the model’s predictions for any given set of model parameters. For termination of the fitting process (and to assess goodness of fit) the tolerance of the error function was set at 10^−4^.

The model was fitted to sex- and age-specific DM (by assuming all DM cases are due to T2DM), obesity, smoking, and physical inactivity prevalence data, as obtained from nationally-representative population-based surveys from Jordan conducted in 1994^12^, 2004^10^, 2007^11^, 2009^14^, and 2017^15^, and one community-based survey conducted in 2004^13^. Thus, the model was fitted to point estimates (but not confidence intervals) of a total of 323 prevalence measures. A brief description of the surveys is in Table 1. The data reflects the resident population of Jordan, but Syrian refugees were not included in the surveys apart from (partially) in the last 2017 survey^15^. Due to substantial differences in response rate across and within surveys, data were weighted during model fitting by survey response rate, and for men and women separately (Table 1). All data used in this study are aggregate, de-identified, and anonymized.

**Table 1:** Characteristics of the Jordan population-based surveys used in the analysis for type 2 diabetes mellitus (T2DM) and its risk factors.

Survey/study title	Survey year	Coverage of data	Age group	Gender distribution		Response rate		Method of diagnosis for diabetes	Risk factors reported	References
				Men	Women	Men (%)	Women (%)			
Diabetes and impaired glucose tolerance in Jordan: prevalence and associated risk factors	1994	National	25+	1046	1790	54	86	Fasting blood glucose greater than 126 mg/dl	Obesity Smoking	^12^
An increase in prevalence of diabetes mellitus in Jordan over 10 years	2004	Sarih in Jordan	25+	394	727	94	94	Either participant had previously been diagnosed with diabetes or had a fasting blood glucose greater than 126 mg/dl	Obesity Smoking	^13^
STEPwise approach to chronic disease risk factor surveillance in 2004	2004	National	18+	1342	1992	85	85	Fasting blood glucose greater than 126 mg/dl	Obesity Smoking	^10^
STEPwise approach to chronic disease risk factor surveillance in 2007	2007	National	18+	1939	1715	86	86	Fasting blood glucose greater than 126 mg/dl	Obesity Smoking	^11^
Relationship between 25-hydroxyvitamin D and metabolic syndrome among Jordanian adults	2009	12 governorates of Jordan	7+	776	2458	36	90	Fasting blood glucose greater than 126 mg/dl	Obesity Smoking	^14^
Time trends in diabetes mellitus in Jordan between 1994 and 2017	2017	National	18+	1099	2495	40	94	Fasting blood glucose greater than 126 mg/dl	Obesity Smoking Physical inactivity	^15^

The model was also fitted to Jordan’s age-specific and total population size (including 4,242 demographic measures) as obtained from the database of the Population Division of the United Nations Department of Economic and Social Affairs (Supplementary Fig. S1)^28^. Due to limited census data for Jordan, we opted to use the United Nations Department of Economic and Social Affairs’ estimates as they provide a more complete picture of the demography and its evolution using a standardized method that is applied globally^28^.

The model was initiated in 1950. The initial conditions for the demographic structure were obtained from the database of the Population Division of the United Nations Department of Economic and Social Affairs^28^. Prevalence of T2DM and related risk factors at initial year were estimated through the fitting process. By generating the best fit, we quantified the following seven rates: sex- and age-specific T2DM baseline incidence rates (i.e. incidences rates from “healthy” to T2DM), and sex- and age-specific transition rates from healthy to obese, obese to healthy, healthy to smoker, smoker to healthy, healthy to physically inactive, and physically inactive to healthy (Table S2).

### Estimating the trends of T2DM and risk factors

Trends in T2DM prevalence and incidence between 1990 and 2050 in the 20–79 years old population of Jordan were generated using the best fit parameters. Trends in risk factors and their effect on T2DM were also predicted for the studied period. The *age-specific* rates of obesity were allowed to increase between 1990 and 2050 (details in Supplementary Information Text S1), to fit the actual trend data^10–15^. Meanwhile, the *age-specific* rates of smoking were assumed to remain constant throughout, as suggested by the actual trend data^10–15^. The *age-specific* rates of physical inactivity were also assumed to remain constant throughout, as only one survey was available for physical inactivity^15^, and hence no trend could be determined. Accordingly, the predicted temporal changes in smoking and physical inactivity prevalence were driven solely by the demographic structure of the population.

The total number of T2DM incident cases attributed to each risk factor was calculated using a methodology developed for estimating the population attributable fraction of risk factors, factoring all of their overlaps^16,29,30^.

### Projections of health expenditure for T2DM

T2DM health expenditure in Jordan was calculated using the Jönsson approach^31^. This approach estimates the health expenditure that is directly attributed to T2DM from the total healthcare expenditure, by converting the *per capita* health expenditure to estimates of T2DM attributable spending. The key parameter for this conversion is the relative ratio of all healthcare expenditure of T2DM individuals compared to non-T2DM individuals ($R_{as}$).

As informed by evidence^32^, we applied two ratios for $$R_{as}$$ (2 and 3) to bracket the range of estimates. We also applied two scenarios for the evolution of Jordan’s *annual per capita* health expenditure over time. First, the *annual per capita* health expenditure was assumed to change between 1990 and 2015, based on historical trend, as reported by World Bank data (Supplementary Fig. S4)^33^, but assumed constant between 2016 and 2050 at the 2015 level ($257 United States dollars^33^). Second, the *annual per capita* health expenditure was assumed to change with time between 1990 and 2050, based on historical trend, as reported by World Bank data^33^, but then also extrapolated into the future based on this trend (Supplementary Fig. S2)^33^. No discounting was applied for cost.

### Sensitivity and uncertainty analyses

We conducted univariate sensitivity analyses to assess robustness of model predictions to variations in the relative risks (RRs) of each risk factor for T2DM incidence, and RR of mortality among people with T2DM compared to the general population. Multivariable uncertainty analyses were also conducted to specify the ranges of uncertainty in projected T2DM prevalence estimates. The first uncertainty analysis was conducted with respect to variations in the key structural model parameters including RR of mortality and RR of developing T2DM if obese, smoker, and physically inactive. The second uncertainty analysis was conducted with respect to variations in each data point of each survey.

For the first uncertainty analysis, Monte Carlo sampling from log-normal distributions was used for the confidence intervals of the epidemiological input parameters (Table S2). Each set of new parameters, generated from the specified ranges, was used to refit the model. Through 1,000 uncertainty runs of the model, the likelihood distribution and the 95% uncertainty interval (UI) for T2DM prevalence were estimated.

For the second uncertainty analysis, Monte Carlo sampling from uniform distributions was used assuming ± 40% uncertainty around each survey data point. Each set of new input data, generated from the specified ranges, was used to refit the model. Through 1000 uncertainty runs of the model, the likelihood distribution and the 95% UI for T2DM prevalence were also estimated.

Finally, we used the regularization technique^34,35^ to investigate whether the model and the fitting method used in this study could be overfitting the existing data. Briefly, by applying this technique additional terms for the fitting parameters were included in the least-square fitting to prevent the fitting parameters from erroneously attaining extreme values^34,35^.

## Results

Supplementary Fig. S1 shows the best-fit of the model to the size of the total population in Jordan as well as to the age-specific demographic distribution. Overall, the model produced good fits for the data, but it did not capture the transient changes in demography due to sudden influx of refugees and immigrants from the year 2010 onwards.

Supplementary Fig. S3 shows the best-fit of the model to the data of the sex- and age-specific T2DM prevalence surveys that were conducted in 1994, 2004, 2009, and 2017, and also to the data of the T2DM STEPwise surveys in 2004 and 2007^10–15^. The best-fit of the model to the various sex- and age-specific prevalence data for obesity, smoking, and physical inactivity are shown in Supplementary Figs. S4-S6. Overall, the model produced good fits for the data, however, for some individual prevalence measures, the model overestimated or underestimated them (Supplementary Figs. S4-S6). These differences may be explained by wider confidence intervals in some of the survey data, or potential biases in the survey measures. For example, in men, non-response rates were very high (Table 1)^10–15^, which may have affected the goodness of fit for some of the measures.

### T2DM prevalence and incidence

**Figure 1:**
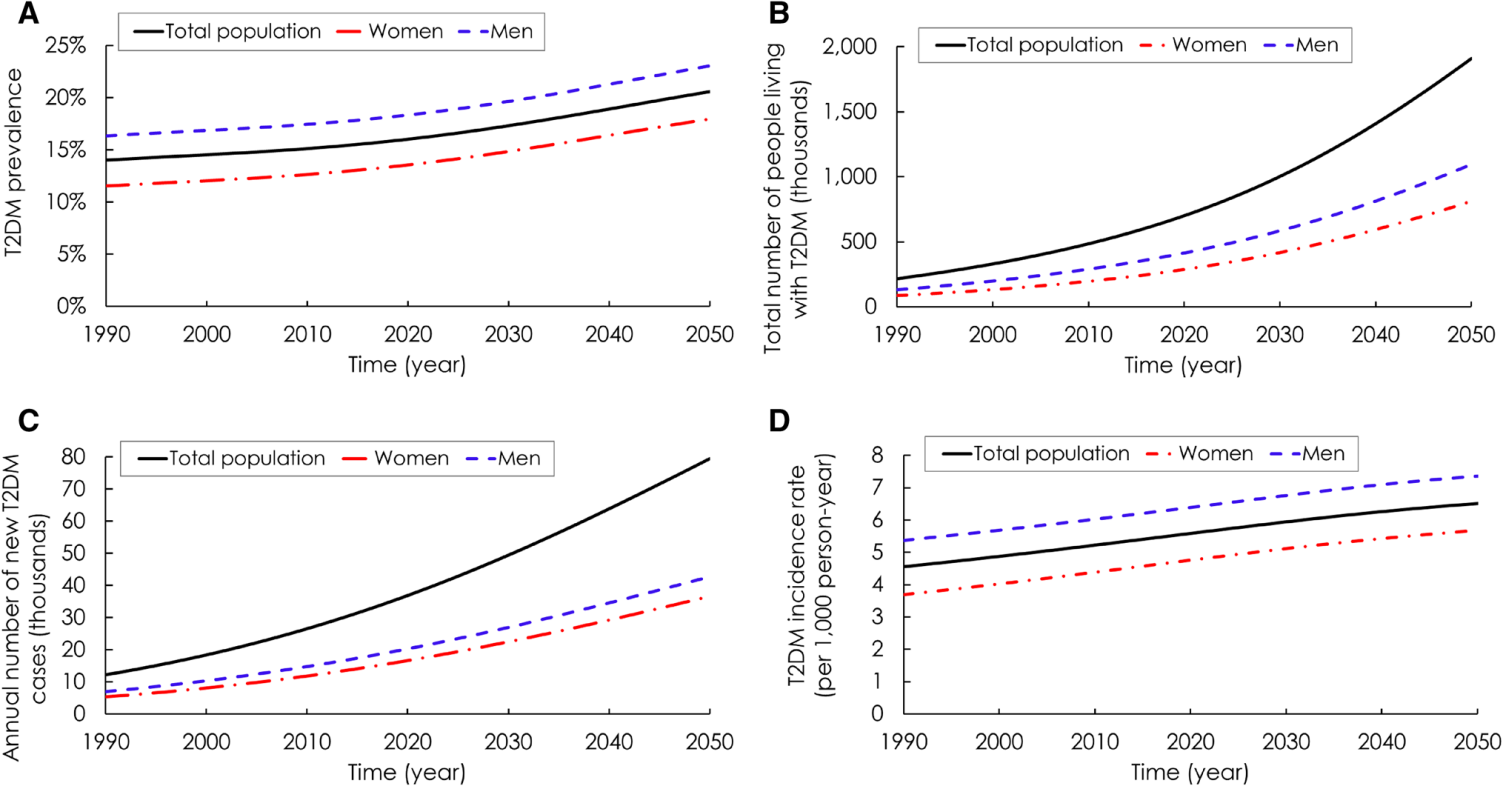
Projected type 2 diabetes mellitus (T2DM) epidemic in Jordan between 1990–2050 in those 20–79 years old. (**A**) Prevalence of T2DM. (**B**) Total number of people living with T2DM. (**C**) Annual number of new T2DM cases. (**D**) T2DM incidence rate.

T2DM prevalence was predicted to increase from 14.0% (95% UI: 13.0–15.0%) in 1990, to 16.0% (95% UI: 14.8–17.0%) in 2020, and to 20.6% (95% UI: 19.2–21.8%) in 2050. T2DM prevalence increased among men from 16.3% in 1990 to 23.1% in 2050, and among women, from 11.5% in 1990 to 18.0% in 2050 (Fig. 1A).

The model projected a steady increase in T2DM incidence (Fig. 1C). In 1990, a total of 218,326 people were living with T2DM (Fig. 1B), of which 12,313 were new cases (Fig. 1C). Incidence rate was predicted to be 4.6 per 1000 person-year (Fig. 1D). In 2020, the total number of T2DM cases were predicted to increase to 702,326 (Fig. 1B), of which 36,941 would be new cases (Fig. 1C). Incidence rate was predicted to be 5.6 per 1000 person-year (Fig. 1D). In 2050, the total number of T2DM cases were predicted to increase further to 1.9 million (Fig. 1B), of which 79,419 would be new cases (Fig. 1C). Incidence rate was predicted to be 6.5 per 1000 person-year (Fig. 1D).

### Health expenditure for T2DM

**Figure 2:**
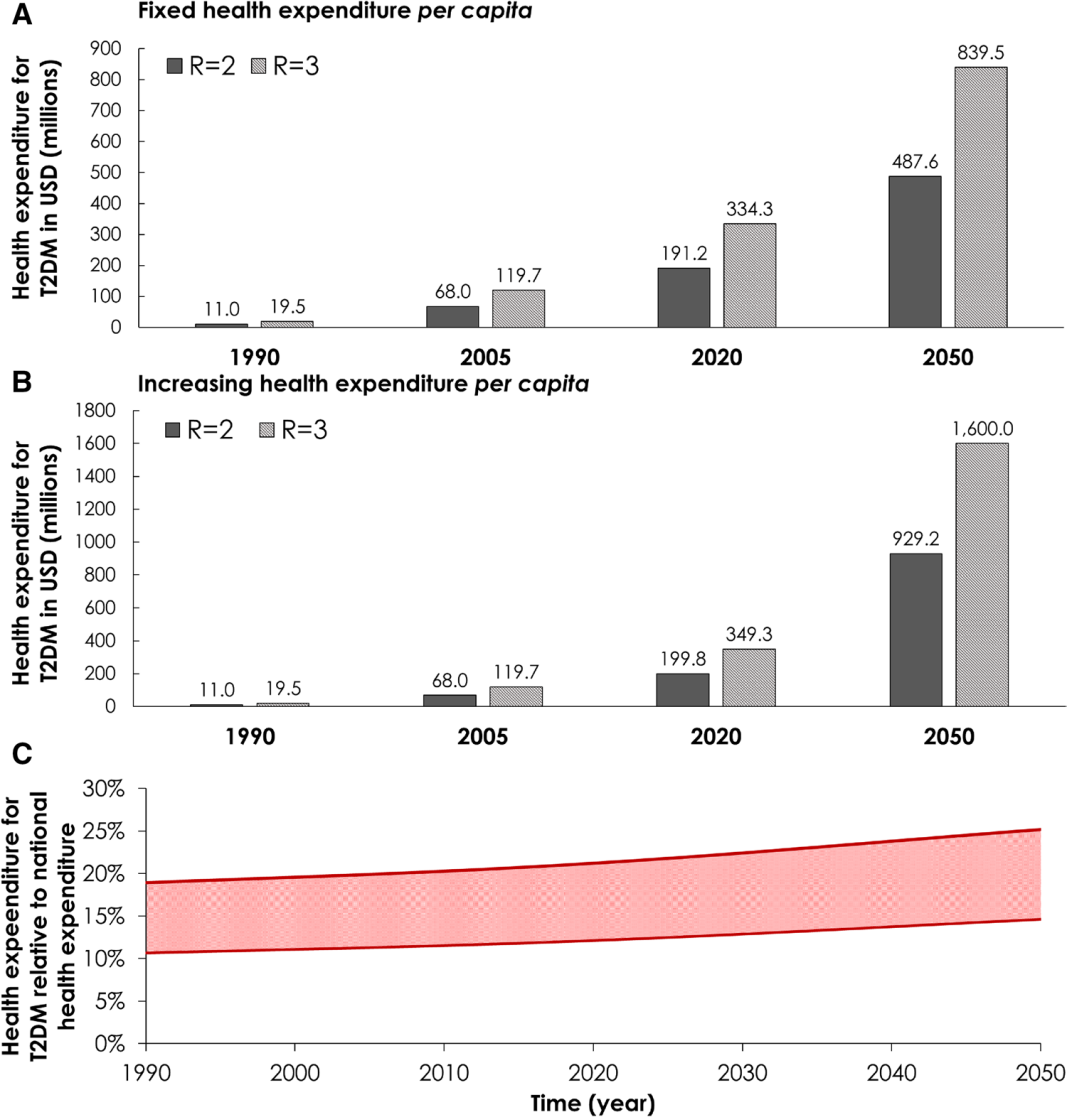
Projected health expenditure for type 2 diabetes mellitus (T2DM) in Jordan, 1990–2050. The figure shows (**A**) expenditure assuming fixed *annual per capita* health expenditure between 2016 and 2050, (**B**) expenditure assuming increasing *annual per capita* health expenditure between 2016 and 2050 based on extrapolation of the increasing historical trend, and (**C**) proportion of Jordan’s total health expenditure that is spent on T2DM. The health expenditure directly attributed to T2DM out of Jordan’s total healthcare expenditure was calculated per the Jönsson’s approach^31^. The *annual per capita* health expenditure in Jordan between 1995 and 2015 was provided by World Bank data^33^.

Figure 2 shows the past, present, and future projected health expenditure for T2DM for $$R_{as} = 2$$ and $$R_{as} = 3$$. In 1990, $50.3–$89.2 were spent per T2DM case resulting in a total expenditure of $11.1–$19.5 million. In this year, T2DM consumed 10.7–19.0% of Jordan’s national health expenditure (Fig. 2C).

Assuming fixed *annual per capita* health expenditure post-2015, $255.4–$439.7 would be spent per T2DM case in 2050 resulting in a total expenditure of $487.6–$839.5 million. In this year, T2DM would consume 14.6–25.2% of national health expenditure (Fig. 2C).

Assuming increasing *annual per capita* health expenditure per extrapolated historical trend, $486.7–$838.0 would be spent per T2DM case in 2050 resulting in a total expenditure of $0.9–$1.6 billion. In this year, T2DM would consume 14.6–25.2% of national health expenditure (Fig. 2C).

### Projections of T2DM-related risk factors

In the 20–79 years old population, prevalence of obesity was predicted to increase from 35.1% in 1990, to 38.0% in 2020, and to 41.4% in 2050 (Supplementary Fig. S8A), while smoking slightly decreased from 27.5% in 1990, to 27.2% in 2020, and to 25.9% in 2050 (Supplementary Fig. S8B). Prevalence of physical inactivity increased from 16.9% in 1990, to 18.2% in 2020, and to 21.2% in 2050 (Supplementary Fig.S8C). The changes in smoking and physical inactivity were only driven by the changes in the demographic structure of the population—the age-specific prevalence of smoking was assumed constant as suggested by trend data^10–15^, while the age-specific prevalence of physical inactivity was assumed constant (as a neutral assumption) due to limited data to inform the trend^15^.

Women had higher obesity prevalence (42.4% in 1990 and 50.9% in 2050) than men (28.3% in 1990 and 33.6% in 2050; Supplementary Fig. S8A). However, women had lower smoking prevalence (9.9% in 1990 and 10.5% in 2050) than men (43.9% in 1990 and 40.5% in 2050; Supplementary Fig. S8B). Women had also lower physical inactivity prevalence (13.3% in 1990 and 17.2% in 2050) than men (20.2% in 1990 and 25.0% in 2050; Supplementary Fig. S8C).

### Proportion of T2DM cases attributable to obesity, smoking, and physical inactivity

**Figure 3:**
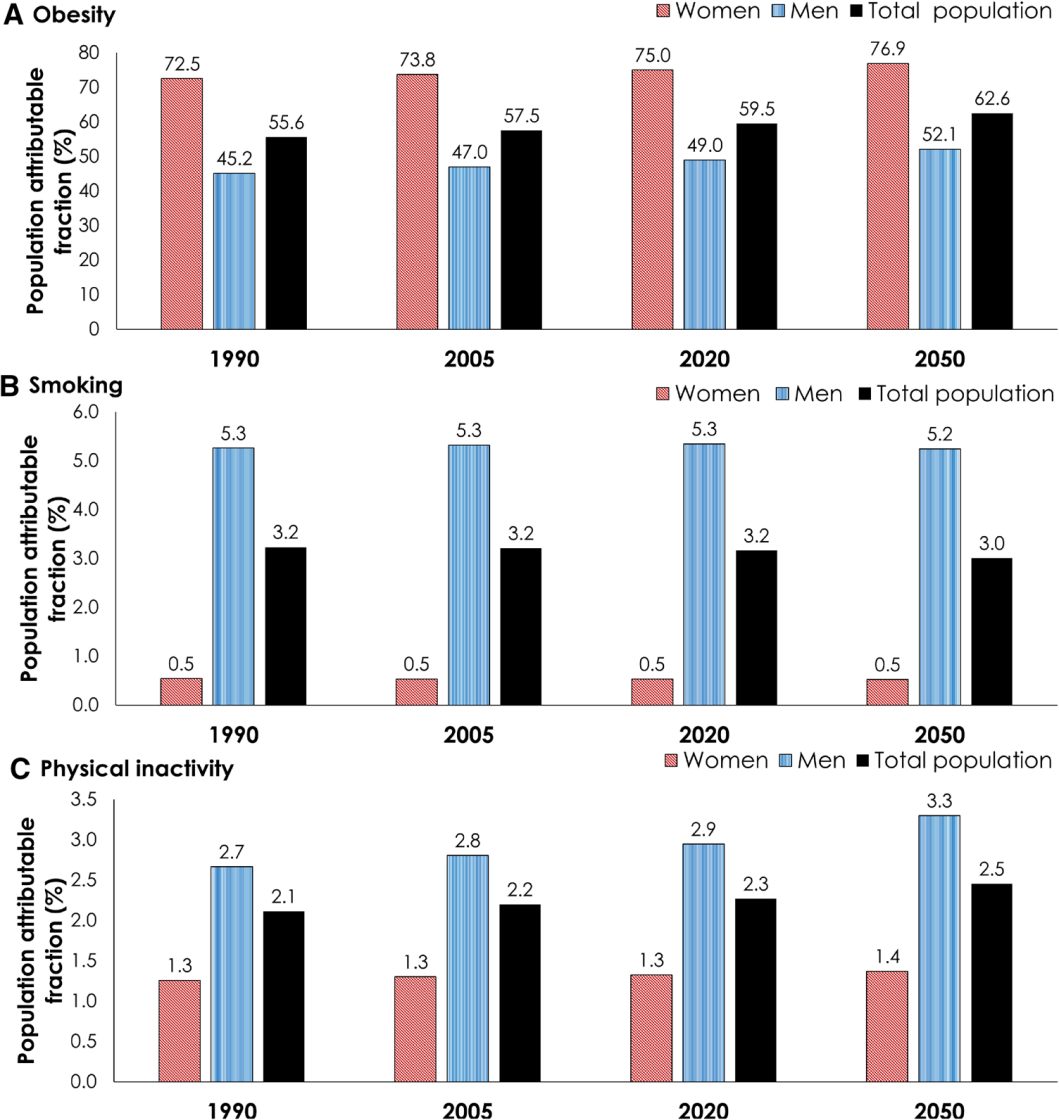
Projections for the proportion of annual new type 2 diabetes mellitus (T2DM) cases in Jordan that is attributed to each key T2DM-related risk factor, 1990–2050. The figure shows projected proportions of T2DM cases attributed to (**A**) obesity, (**B**) smoking, and (**C**) physical inactivity.

Figure 3 shows the predictions for the proportions of incident T2DM cases that are attributed to each of obesity, smoking, and physical inactivity at four different time points between 1990 and 2050. The proportion of annual new T2DM cases attributed to obesity increased from 55.6% in 1990, to 59.5% in 2020, and to 62.6% in 2050 (Fig. 3A). This proportion increased among men from 45.2% in 1990 to 52.1% in 2050, and among women, from 72.5% in 1990 to 76.9% in 2050 (Fig. 3A).

The proportion of incident T2DM cases attributed to smoking was constant at 3.2% between 1990 and 2020, but decreased slightly to 3.0% in 2050 (Fig. 3B). This proportion decreased among men from 5.3% in 1990 to 5.2% in 2050, but was constant among women at 0.5% between 1990 and 2050 (Fig. 3B).

The proportion of incident T2DM cases attributed to physical inactivity increased from 2.1% in 1990, to 2.3% in 2020, and to 2.5% in 2050 (Fig. 3C). This proportion increased among men from 2.7% in 1990 to 3.3% in 2050, and among women, from 1.3% in 1990 to 1.4% in 2050 (Fig. 3C).

### Sensitivity and uncertainty analyses

The robustness of the findings was assessed in sensitivity analyses (Supplementary Fig.S7). In 2050, T2DM prevalence in the 20–79 years old population ranged from 18.4–23.2%, 20.4–20.8%, and was largely unaltered at 20.6% by varying each RR of developing T2DM if obese, smoker, and physically inactive, respectively. T2DM prevalence ranged from 20.0 to 21.4% by varying the RR of mortality in T2DM compared to the general population.

The range of uncertainty in the projected T2DM prevalence estimates was assessed in two uncertainty analyses (Supplementary Fig.S9A and S9B). The UIs around the trends in T2DM prevalence between 1990 and 2050 were relatively narrow highlighting that the predictions were consistent to a wide range of parameter assumptions and input data errors or variations.

Finally, the robustness of the findings was also investigated using the regularization technique^34,35^ (Supplementary Fig.S9C). Though T2DM prevalence was slightly higher after regularization, it was within the reported UIs of the uncertainty analyses (Supplementary Fig. S9A and S9B). Moreover, the trend in T2DM prevalence was invariable highlighting the reliability and robustness of our predictions (Supplementary Fig.S9C).

## Discussion

We provided a characterization of the T2DM epidemic in Jordan. T2DM prevalence increased by 14.3% between 1990 and 2020 and was projected to increase further by 28.8% between 2020 and 2050. In 2020, as much as $350 million would be spent on T2DM, consuming 21% of the national health expenditure. By 2050, T2DM could consume about 25% of the national health expenditure. The epidemic was found to be largely driven by obesity, contributing about 60% of T2DM incidence. The role of obesity was also more pronounced for women than men, reflecting the high prevalence in women that is consistent and observed in the region^36^. While smoking and physical inactivity played a role in the expanding epidemic, their contribution was small compared to obesity—both combined contributed only about 5% of T2DM incidence. These findings demonstrate that the epidemic is largely a reflection of modifiable risk factors, namely obesity, and is best addressed through appropriate lifestyle interventions that improve the health profile of the population.

Demography was found to play a major role in the epidemic expansion over the next three decades. The population of Jordan was projected to grow by 89% between 2020 and 2050, while the mean age of the population would increase by seven years, from 26 to 33 years. The coupling of increasing population size with progressive aging demonstrates how difficult it will be to halt or control the epidemic. The number of people living with T2DM was projected to rapidly increase from 218,326 in 1990, to 702,326 in 2020 and 1.9 million in 2050, swiftly straining an already resource-strained healthcare system. Jordan is all but destined to confront an immense healthcare challenge and an escalating health expenditure that it may not be able to afford.

Evidence indicates that policy-level facilitation is essential to yield an environment that nudges people to make the right choices to reduce their lifestyle risk factors, and thus T2DM risk^37–39^. Studies have shown that structural interventions targeting T2DM or its risk factors have the potential to change T2DM epidemiology and impact the epidemic^37,38,40^. Such structural interventions are often fiscal, legislative, or environmental in nature, and outside the immediate control of the individual or the health sector. They include policies to reduce consumption of unhealthy foodstuffs through fiscal regulations (e.g. increasing taxation on sugar-sweetened beverages), or subsidies for healthier foods (e.g. fruits and vegetables)^40^. However, the feasibility and political palatability of such approaches in Jordan require further consideration and investigation.

Other studies have projected the burden of T2DM in Jordan. The International Diabetes Federation (IDF)^41^ estimated that T2DM prevalence for those aged 20–79 was 9.5% in 2017 and will increase to 12.5% in 2045—an increase of 31.6%^41^. Though the prevalence of T2DM was projected to be higher in our study, the projected increase between 2017 and 2045 was lower than that reported by IDF. Our study predicted an increase of 26%, from 15.7% in 2017 to 19.8% in 2045. Differences in the T2DM levels and projections are primarily due to differences in the surveys used as input data—we used more (and all available) population-based survey data to power the modelling projections. Differences are also due to the implemented modelling methodology. To estimate T2DM levels, IDF used a logistic regression method rather than a dynamical population-level model of T2DM and its key risk factors, as is the case in the present study. Of note that the characterized T2DM epidemic in Jordan is similar in scale and epidemiology to the epidemics seen and characterized in other MENA countries, such as Lebanon^2^, Saudi Arabia^2,42^, Tunisia^2,43^, and Qatar^2,16^. However, when compared to Palestine and Syria^2^, our estimates were somewhat larger than those estimated in the IDF report for these two countries, but similar to those estimated in other modelling studies^44,45^. This demonstrates the largely regional nature of these T2DM epidemics.

Our study has limitations. The modelling predictions hinge on the representativeness of the input data, particularly the survey data. T2DM surveys in Jordan varied in study design, specific definition of outcomes, survey administration, and response rate, thus potentially biasing measures of T2DM and related risk factors. In most surveys, the non-response rate in men was substantially higher than in women (Table 1)^10–15^—included men may not have been representative of the wider population of men. For instance, physical *inactivity* was higher in men than women, contrary to expectation, especially considering that the prevalence of obesity was higher in women^10–15^. This could be a biased result due to the sex differences in response rate. Also, body mass index ≥ 30 kg/m^2^ was used to define obesity because it is a pragmatic measure in surveys^46^. But this may not be the most representative measure to capture the impact of obesity on T2DM incidence^46^. Given also the differences in body fat distribution between men and women^46^, we may have underestimated the impact of obesity on T2DM among men. T2DM prevalence in men was substantially higher than in women.

Although T2DM prevalence in men was expected to be higher, the relative difference between men and women was larger than expected. This difference is driven by the difference observed in the actual country-specific survey data used to calibrate the model^10–15^, and seems consistent with the differences observed in other countries^47^. Moreover, given that the T2DM surveys in Jordan varied in study design and methods and were conducted at different time points thus potentially introducing bias, fitting all the survey data simultaneously with the ordinary least-square estimator could possibly lead to overfitting that affects the results. Nonetheless, a regularization technique was applied to investigate overfitting and the results affirmed our findings and conclusions (Supplementary Fig.S9C).

Another limitation in this study is the lack of published Jordan-specific data on the relative ratio of healthcare expenditure in T2DM individuals versus non-T2DM individuals. To be consistent with global empirical evidence^31^, we used the conventional range of 2–3 to bracket the estimates of the T2DM economic burden. Similarly, although the estimates of the RRs of T2DM with respect to the risk factors were obtained from large, quality prospective studies that are pooled through global systematic reviews and meta-analyses^4,7,9^, the representativeness of these RRs for the population of Jordan remains unknown. We did not formally investigate identifiability in this model, nor in this class of models. Unlike other disciplines, such as within host modelling and biological systems modelling^48–50^, there is limited research on identifiability for disease population-level models, particularly for diabetes^51^. Of note, however, that our model does not have complex non-linearities as in infectious disease models. Our focus also is not on parameter estimation, but on capturing the epidemiologic trends. Therefore, it is less likely that this class of models could be affected by serious identifiability issues. We did not *explicitly* include the waves of refugees and immigrants hosted by Jordan due to the political conflicts surrounding Jordan over the past few decades. Consequently, some dynamics (or sudden changes in the population) reported in the United Nations projections were not explicitly accounted for in the fitting of the demographic indicators.

Nonetheless, this study has several strengths. This mathematical modelling work was anchored on nationally-representative population-based data of different surveys at different times—a total of six surveys. Model calibration ensured that a best fit of input data is attained, adjusting for discrepancies in data input and accounting for the level of confidence in each datapoint. This highlights the power of such modelling approach to characterize T2DM epidemiology. As survey outcome measures often vary due to survey quality, time, design, geographic coverage, outcome definitions, selection and information biases, and differentials in response rate^52–56^, this approach “reconciles” the differences in survey data by producing the most consistent characterization of the underlying epidemiology. For example, Jordan surveys are limited by sex-differentials in response rate (Table 1)^10–15^. The model fitting, however, has adjusted for the level of confidence in each survey data by weighting each datapoint differentially by the response rate. Moreover, to account for potential variations or errors in each data point of each survey, an uncertainty analysis was conducted and the results affirmed our findings and conclusions (Supplementary Fig. S9B).

Moreover, we conducted several sensitivity analyses to assess the robustness of our predictions (Supplementary Fig.S7). The analyses demonstrated that while our results are sensitive to the T2DM-obesity RR, they are insensitive to variations in the remaining parameters. We further conducted multivariate uncertainty analyses that demonstrated narrow UIs around the point estimates (Supplementary Figs. S9A and S9B). Therefore, both the sensitivity and uncertainty analyses affirm the robustness of our predictions.

In conclusion, Jordan is confronted with a rapidly rising T2DM epidemic over the coming decades. With a progressively aging population and ever-increasing obesity prevalence, T2DM incidence and prevalence will continue to grow despite already reaching high levels. Obesity is the driver of half of T2DM incidence, highlighting the unique contribution of this risk factor to this major public health challenge. With the growing epidemic, T2DM health expenditure will escalate to account for a quarter of Jordan’s health expenditure. These findings demonstrate the criticality of mass national engagement and large-scale implementation of cost-effective preventive and therapeutic interventions targeting T2DM and its risk factors.

## Supplementary information

Supplementary Information.

## Data Availability

Data are available in the cited literature, main manuscript, and Supplementary Information. The codes programmed in MATLAB can be obtained by contacting the authors.
