# Supplementary material for: Characterizing the type 2 diabetes mellitus epidemic in Jordan up to 2050
Source: Sci Rep. 2020 Dec 3;10:21001. doi: 10.1038/s41598-020-77970-7 (PMC7713435; doi:10.1038/s41598-020-77970-7)
Supplement: Supplementary file 1 — Supplementary Information. [file 41598_2020_77970_MOESM1_ESM.docx]

**Supplementary Information**

**Characterizing the type 2 diabetes mellitus epidemic in Jordan up to 2050**

Susanne F. Awad,^1,2,3^ Peijue Huangfu,^4^ Soha R. Dargham,^1,2^ Kamel Ajlouni,^5^ Anwar Batieha,^6^ Yousef S. Khader,^6^ Julia A. Critchley,^4^ and Laith J. Abu-Raddad^1,2,3^

^1^Infectious Diseases Epidemiology Group, Weill Cornell Medical College – Qatar, Cornell University, Doha, Qatar

^2^World Health Organization Collaborating Centre for Disease Epidemiology Analytics on HIV/AIDS, Sexually Transmitted Infections, and Viral Hepatitis, Weill Cornell Medicine – Qatar, Doha, Qatar

^3^Department of Population Health Sciences, Weill Cornell Medicine, Cornell University, New York, USA

^4^Population Health Research Institute, St George’s, University of London, London, UK

^5^The National Centre for Diabetes, Endocrine and Genetics, The University of Jordan, Amman, Jordan

^6^Department of Public Health and Community Medicine, Jordan University of Science and Technology, Irbid, Jordan

**Text S1—Further details on the methods**

The model is an adaptation of an earlier type 2 diabetes mellitus (T2DM) mathematical model.^1^ The mathematical expressions, the main changes to the original model, and the parameters’ description are described below. The model was programmed in MATLAB version 2018b.^2^

We described the T2DM natural history in this age-structured model by the progression states of susceptible that were stratified by 1) healthy, 2) obese, 3) smoker, 4) physically inactive, 5) obese and smoker, 6) obese and physically inactive, 7) smoker and physically inactive, and 8) obese, smoker, and physically inactive; and by the progression states of T2DM disease that were also stratified by the same risk factor states and their overlap. The population was further divided into distinct age and sex groups.

*Susceptible population with up to one risk factor*

We assumed that individuals were born “healthy” susceptible—meaning that they did not have T2DM nor T2DM-related risk factors. Individuals remained in the “healthy” state until they became obese, active smokers, physically inactive, or progressed to T2DM. Individuals in any of these susceptible states were assumed to die of natural causes (i.e. causes not related to T2DM).

Those in the “obese” state remained as such until they became smokers (i.e. moved to the overlapping compartment of “obese smoker”), physically inactive (i.e. moved to the overlapping compartment of “obese physically inactive”), “healthy” again (i.e. became non-obese), or progressed to T2DM. Those in the “smoker” state remained as such until they became obese, physically inactive, “healthy” again, or progressed to T2DM. Those in the “physical inactivity” state remained as such until they became obese, smokers, “healthy” again, or progressed to T2DM.

*Susceptible population with overlap of more than one risk factor (for those >4 years old)*

Individuals in the “obese smoker” state remained as such until they became physically inactive (i.e. moved to the overlapping compartment of “obese, smoker, physically inactive”), moved to “obese” state, moved to “smoker” state, or developed T2DM. Those in the “obese physically inactive” state remained as such until they became smokers, moved to “obese” state, moved to “physically inactive” state, or developed T2DM. Those in the “smoker physically inactive” state remained as such until they became obese, moved to “smoker” state, moved to “physically inactive” state, or developed T2DM. Individuals in the “obese, smoker, physically inactive” state remained as such until they moved to “obese smoker”, “obese physically inactive”, or “smoker physically inactive”, or developed T2DM.

*Populations with T2DM with up to one or more risk factors (for those >4 years old)*

Individuals with T2DM remained diabetic (i.e. assuming there was no remission), or died of natural or disease-related causes. T2DM individuals with one risk factor could develop the second risk factor, or reverse to T2DM with none of the risk factors. Those with two risk factors could develop a third risk factor, or reverse to only one of the risk factors, while those with three risk factors could reverse one of the current risk factors.

Definitions of all symbols in the equations of the model can be found in Tables S1.

**Table S1. Definitions of the symbols in the equations of the type 2 diabetes mellitus (T2DM) age-structured mathematical model.**

| **Symbol** | **Definition** |
| --- | --- |
| $H_{a}$ | “Healthy” T2DM-susceptible population (do not have T2DM nor T2DM-related risk factors) |
| $O_{a}$ | T2DM-susceptible but obese population^#^ |
| $S_{a}$ | T2DM-susceptible but smoker population |
| $F_{a}$ | T2DM-susceptible but physically inactive population |
| $OS_{a}$ | T2DM-susceptible but obese and smoker population |
| $OF_{a}$ | T2DM-susceptible but obese and physically inactive population |
| $SF_{a}$ | T2DM-susceptible but smoker and physically inactive population |
| $OSF_{a}$ | T2DM-susceptible but obese, smoker, and physically inactive population |
| $DM_{\iota}$ | Populations with T2DM where the index $\iota$ marks the risk factor status; $\iota=H, O,\text{ }S, \text{ }F, \text{ }OS, \text{ }OF, \text{ }SF,\text{ }OSF$ |
| $N$ | Total population size |
| $\varsigma$ | Transition rate from one age group ($a$) to the next age group |
| $\lambda_{\iota\to DM_{\iota}}$ | T2DM-disease progression rate where the index $\iota$ marks the risk factor status; $\iota=H, O,\text{ }S, \text{ }F, \text{ }OS, \text{ }OF, \text{ }SF,\text{ }OSF$ |
| $\mu_{a}$ | Natural death rate |
| $cf_{a}$ | T2DM-related death rate |
| $RR_{\iota}$ | Relative risk of developing T2DM where the index $\iota$ marks the risk factor status; $\iota=H, O,\text{ }S, \text{ }F, \text{ }OS, \text{ }OF, \text{ }SF,\text{ }OSF$ |
| $\alpha_{a}$**, $\beta_{a}$,** $\Im_{a}$ | Transition rates from “healthy” (regardless of T2DM status) with none of the risk factors to one of the risk factors; i.e. becomes obese ($O$), smoker ($S$), or physically inactive ($F$) |
| $\nu_{a}$**, $\eta_{a}$, $\chi_{a}$,$\omega_{a}$, $\xi_{a}$,**$\psi_{a}$ | Transition rates from having one of the risk factors to having two risk factors (i.e. becomes $OS$, $OF$, or $SF$; regardless of T2DM status) |
| $\sigma_{a}$**, $\delta_{a}$,** | Transition rates from having one of the risk factors to being “healthy” with none of the risk factors (regardless of T2DM status) |
| $\kappa_{a}$**, $Є_{a}$,** $\Omega_{a}$ | Transition rates from having two of the risk factors to having three of the risk factors (regardless of T2DM status) |
| $\varepsilon_{a}$**, $\gamma_{a}$,**  **,**  $\theta_{a}$ $\pi_{a}$**,** $\rho_{a}$ | Transition rates from having two of the risk factors to having one of the risk factors (regardless of T2DM status) |
| **, $o_{a}$,** $\upsilon_{a}$ | Transition rates from having three of the risk factors to having two of the risk factors (regardless of T2DM status) |

*^#^* Defined as body mass index >30 kg per m^2^.^3^

Due to the nature of available data, the following changes were necessary in the present work relative to our previous study:^1^

*Population growth and mortality rates*

The population growth rate ($b(t)$) and the natural mortality rate ($\mu(t,a)$) were described by the following functions,^4^ providing a good fit of the population growth and demographic age structure in Jordan:^5^

$$b(t)=a_{0}e^{-\left(\frac{t-t_{0}}{b_{0}}\right)^{2}}$$

and

$$\mu(t,a)=\frac{a_{1}e^{-\left(\frac{t-t_{1}}{b_{1}}\right)^{2}}}{\left[1+e^{-b_{2}\left(a-a_{2}\right)}\right]}$$

Here, the parameters $a_{0}$, $a_{1}$, $a_{2}$, $t_{0}$, $t_{1}$, $b_{0}$, $b_{1}$, and $b_{2}$were obtained by fitting the model to the demographic data of Jordan from the database of the Population Division of the United Nations Department of Economic and Social Affairs.^5^

*Obesity onset rate*

Given evidence for increasing obesity prevalence in Jordan, the rate of becoming obese in the T2DM model was allowed to be time- and age-dependent and was parameterized through a combined Gaussian-logistic function:

$\alpha(t,a)=\frac{c_{1}e^{-\left(\frac{t-t_{2}}{d_{1}}\right)^{2}}}{\left[1+e^{-d_{2}\left(a-c_{2}\right)}\right]}$.

Here, $c_{1}$, $c_{2}$, $t_{2}$, $d_{1}$, and $d_{2}$ are fitting parameters obtained by fitting the model to the age-structured obesity prevalence data.^6-11^

**Text S2—Data sources**

The T2DM model was parameterized using empirical epidemiological and natural history data from multiple sources. The parameters’ definitions and data sources related to the T2DM model are in Table S2, while the model’s parameter values along with their references are listed in Table S3.

Table S2. Parameters’ definitions and sources for the input data of the type 2 diabetes mellitus (T2DM) model.

| Symbol | Parameter | Reference | Range used for uncertainty or sensitivity analyses or additional information |
| --- | --- | --- | --- |
| $\varsigma$ | Transition rate from one $a_{i}$ to $a_{i+1}$ | - | - |
| $a$ | Number of age compartments in the model | - | - |
| $RR_{O}$ | Relative risk of developing T2DM if obese | 12 | Based on reported confidence interval (CI) |
| $RR_{S}$ | Relative risk of developing T2DM if current smoker | 13 | Based on reported CI |
| $RR_{F}$ | Relative risk of developing T2DM if physically inactive | 14 | Based on reported CI |
| $RR_{OS}$ | Relative risk of developing T2DM if obese and smoker | Calculated* based on ^12,13^ | Calculated based on CI in ^12,13^ |
| $RR_{OF}$ | Relative risk of developing T2DM if obese and physically inactive | Calculated* based on ^12,14^ | Calculated based on CI in ^12,14^ |
| $RR_{SF}$ | Relative risk of developing T2DM if smoker and physically inactive | Calculated* based on ^13,14^ | Calculated based on CI in ^13,14^ |
| $RR_{OSF}$ | Relative risk of developing T2DM if obese, smoker, and physically inactive | Calculated* based on ^12-14^ | Calculated based on CI in ^12-14^ |
| $RR_{M}$ | RR of mortality in T2DM as compared to the general population | 15,16 | ±25% |
| $R_{as}$ | The ratio of medical expenditures for individuals with T2DM relative to individuals without T2DM | 17 | - |
| Country specific variables | | | |
| $\alpha_{H\to O}$ | Transition rates from healthy to obese | Obtained by fitting the model to available data on prevalence of obesity from national and regional surveys.^6-8,10,11^ | Fitting parameter |
| $\sigma_{O\to H}$ | Transition rates from obese to healthy |  | Fitting parameter |
| $\beta_{H\to S}$ | Transition rates from healthy to smoker | Obtained by fitting the model to available data on prevalence of smoking from national and regional surveys.^6-8,10,11^ | Fitting parameter |
| $\delta_{S\to H}$ | Transition rates from smoker to healthy |  | Fitting parameter |
| $\Im_{H\to F}$ | Transition rates from healthy to physically inactive | Obtained by fitting the model to available data on prevalence of physical inactivity from a national survey.^11^ | Fitting parameter |
|  | Transition rates from physically inactive to healthy |  | Fitting parameter |
| $\lambda_{H\to DM_{H}}$ | T2DM baseline incidence rate (i.e., incidence rate from “healthy” to T2DM) | Obtained by fitting the model to available data on T2DM prevalence from national and regional surveys. ^6-11^ | Fitting parameter |
| $\lambda_{O\to DM_{O}}$ | T2DM incidence rate among obese | Calculated based on $RR_{O}\times\lambda_{H\to DM_{H}}$ | Fitting parameter |
| $\lambda_{S\to DM_{S}}$ | T2DM incidence rate among smoker | Calculated based on $RR_{S}\times\lambda_{H\to DM_{H}}$ | Fitting parameter |
| $\lambda_{F\to DM_{F}}$ | T2DM incidence rate among physically inactive | Calculated based on $RR_{F}\times\lambda_{H\to DM_{H}}$ | Fitting parameter |
| $\lambda_{OS\to DM_{OS}}$ | T2DM incidence rate among obese and smoker | Calculated based on $RR_{OS}\times\lambda_{H\to DM_{H}}$ | Fitting parameter |
| $\lambda_{OF\to DM_{OF}}$ | T2DM incidence rate among obese and physically inactive | Calculated based on $RR_{OF}\times\lambda_{H\to DM_{H}}$ | Fitting parameter |
| $\lambda_{SF\to DM_{SF}}$ | T2DM incidence rate smoker and physically inactive | Calculated based on $RR_{SF}\times\lambda_{H\to DM_{H}}$ | Fitting parameter |
| $\lambda_{OSF\to DM_{OSF}}$ | T2DM incidence rate among obese, smoker, and physically inactive | Calculated based on $RR_{OSF}\times\lambda_{H\to DM_{H}}$ | Fitting parameter |
| $N$ | Total population | For each year per the database of the Population Division of the United Nations Department of Economic and Social Affairs.^5^ | - |
| $b$ | birth rate | Obtained by fitting the model to available data on population structure and size from the Population Division of the United Nations Department of Economic and Social Affairs.^5^ | Fitting parameter |
| $\mu$ | Natural mortality rate | Obtained by fitting the model to available data on population structure and size from the Population Division of the United Nations Department of Economic and Social Affairs.^5^ | Fitting parameter |

*Assumed a multiplicative association for the overlapping risk groups.

**Table S3. Model assumptions in terms of parameter values.**

| ***Assumption*** | ***Age group*** | ***Parameter value (95% CI)*** | | ***Reference*** |
| --- | --- | --- | --- | --- |
|  |  | **Men** | **Women** |  |
| Number of age compartments in the model (each for 5 years; *a*) | - | 20 | 20 | - |
| Relative risk of developing T2DM if obese ($RR_{O}$) | All | 6.48 (5.17–8.13) | 8.38 (5.46–12.85) | 12 |
| Relative risk of developing T2DM if current smoker ($RR_{S}$) | All | 1.42 (1.34–1.50) | 1.33 (1.26–1.41) | 13 |
| Relative risk of developing T2DM if physically inactive ($RR_{F}$) | 15–69  70–79  ≥80 | 1.45 (1.37–1.54)  1.32 (1.25–1.40)  1.20 (1.14–1.28) | 1.45 (1.37–1.54)  1.32 (1.25–1.40)  1.20 (1.14–1.28) | 14 |
| Relative risk of developing T2DM if obese and smoker ($RR_{OS}$) | All | 9.20 (6.93–12.20) | 11.15 (6.88–18.12) | Calculated based on ^12,13^ |
| Relative risk of developing T2DM if obese and physically inactive ($RR_{OF}$) | 15–69  70–79  ≥80 | 9.40 (7.08–12.52)  8.55 (6.46–11.38)  7.78 (5.89–10.41) | 12.15 (7.48–19.79)  11.06 (6.83–18.12)  10.06 (6.22–16.45) | Calculated based on ^12,14^ |
| Relative risk of developing T2DM if smoker and physically inactive ($RR_{SF}$) | 15–69  70–79  ≥80 | 2.06 (1.84–2.37)  1.87 (1.68–2.17)  1.70 (1.53–1.97) | 1.93(1.73–2.17)  1.76 (1.58–1.99)  1.60 (1.44–1.80) | Calculated based on ^13,14^ |
| Relative risk of developing T2DM if obese, smoker, and physically inactive ($RR_{OSF}$) | 15–69  70–79  ≥80 | 13.34 (9.49–19.28)  12.15 (8.66–17.65)  11.04 (7.90–16.03) | 16.16 (9.43–27.90)  14.71 (8.60–25.55)  13.37 (7.84–23.19) | Calculated based on ^12-14^ |
| RR of mortality in T2DM as compared to the general population ($RR_{M}$) | 20–29  30–39  40–49  50–59  60–69  70–79+ | 3.70  3.30  1.95  1.65  1.62  1.40 | 5.95  5.61  3.41  2.73  2.08  1.78 | 15,16 |
| The ratio of medical expenditure for individuals with T2DM relative to individuals without T2DM ($R_{as}$) | All | 2  3 | 2  3 | 17 |
| Jordan’s health expenditure per capita (United States dollars) | All | 358.91 | 358.91 | 18 |

**Additional Figures**

**Figure S1.** Model prediction for **A)** the population size of Jordan, **B)** the proportion of the population in each age-group in 2020, **C)** the proportion of the population in each age-group in 2050, and **D)** the mean age of the population. Model predictions in panels A-C were compared to the estimates of the Population Division of the United Nations Department of Economic and Social Affairs.^5^

**
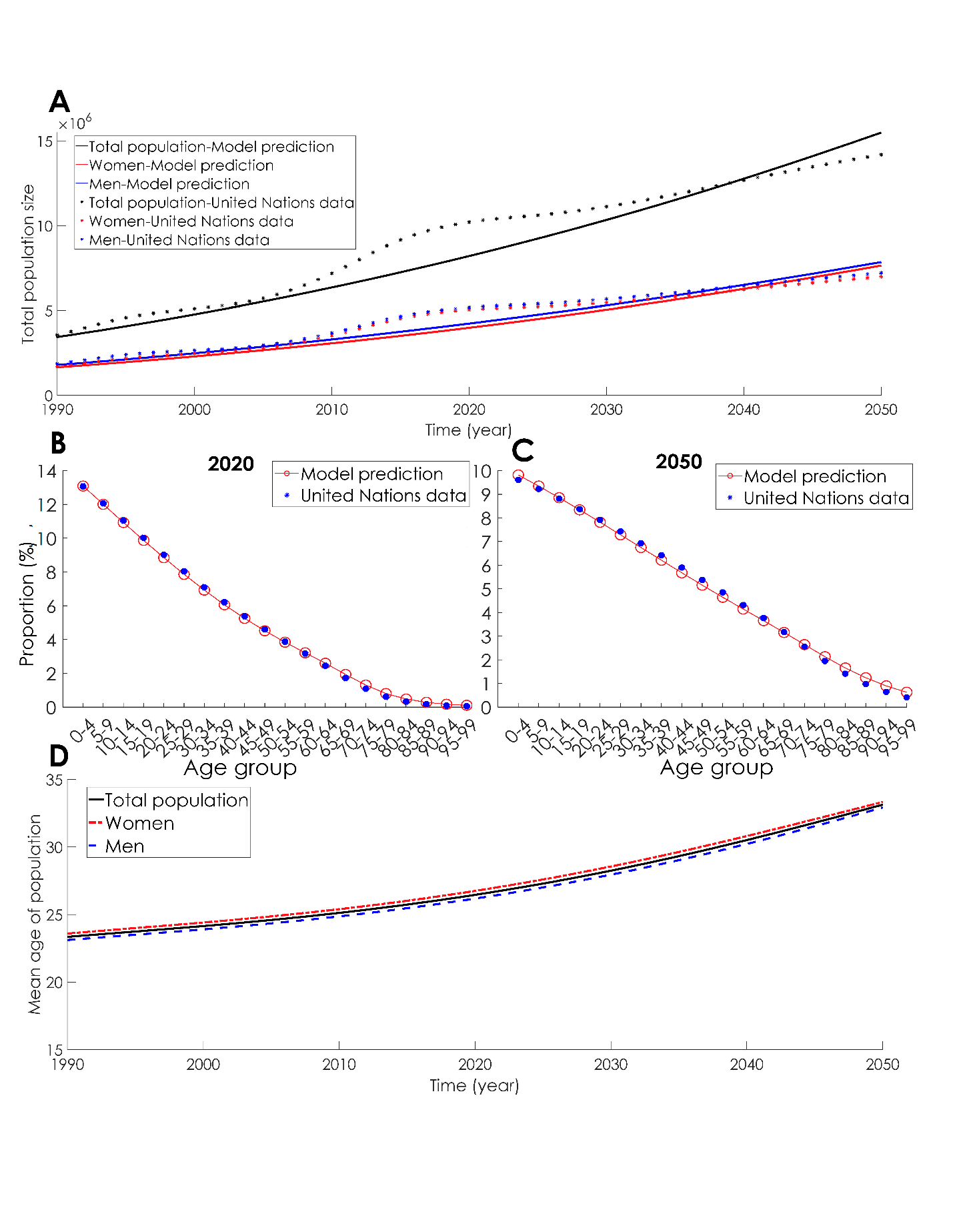
**

**Figure S2.** Trend in *annual per capita* health expenditure (in United States dollars; USD) in Jordan between 1995-2015, as reported by World Bank data.^18^

**
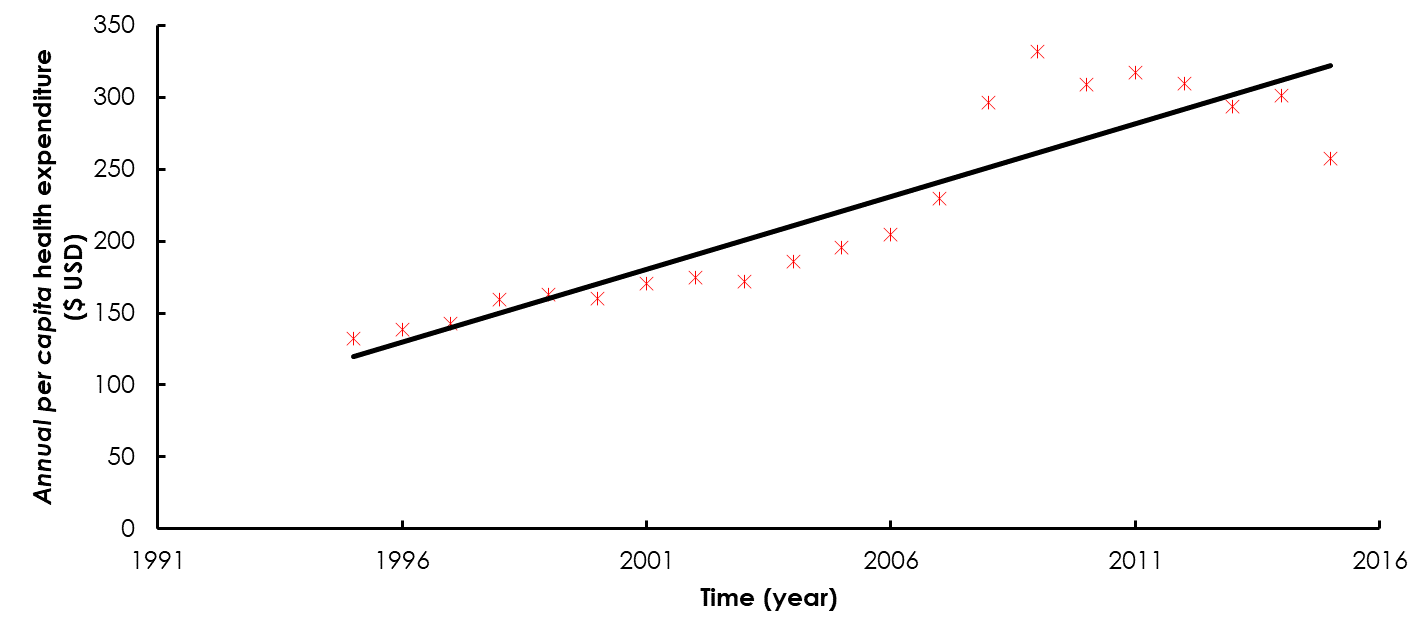
**

**Figure S3.** Model fit for the survey sex- and age-specific type 2 diabetes mellitus (T2DM) prevalence in Jordan in 1994 (panels A and B), 2004 (panels C and D), 2009 (panels E and F), and 2017 (panels G and H), and also for the 2004 (panel I) and 2007 (panel J) STEPwise surveys. The black crosses in the panels are the data provided by the different population-based surveys in these years.^6-11^

**Figure S4.** Model fit for the survey sex- and age-specific obesity prevalence in Jordan in 1994 (panels A and B), 2009 (panels C and D), and 2017 (panels E and F), and also for the 2004 (panel G) and 2007 (panel H) STEPwise surveys. The black crosses in the panels are the data provided by the different nationally-representative population-based surveys in these years.^6-8,10,11^

**Figure S5.** Model fit for the survey sex- and age-specific smoking prevalence in Jordan in 1994 (panels A and B), 2009 (panels C and D), and 2017 (panels E and F), and also for the 2004 (panel G) and 2007 (panel H) STEPwise surveys. The black crosses in the panels are the data provided by the different nationally-representative population-based surveys in these years.^6-8,10,11^

**Figure S6.** Model fit for the survey sex- and age-specific physical inactivity prevalence in Jordan in 2017. The black crosses in the panels are the data provided by the nationally-representative population-based survey of 2017.^11^

**Figure S7.** Sensitivity analyses to assess the sensitivity of the model predictions for type 2 diabetes mellitus (T2DM) prevalence in 2050 to variations in the relative risks (RRs) of the T2DM-related risk factors and RR of mortality in T2DM compared to the general population. Blue bars are based on the lower bound of parameter values (lower bound of the 95% confidence interval; CI) and orange bars are based on the upper bound of parameter values (upper bound of the 95% CI).

**
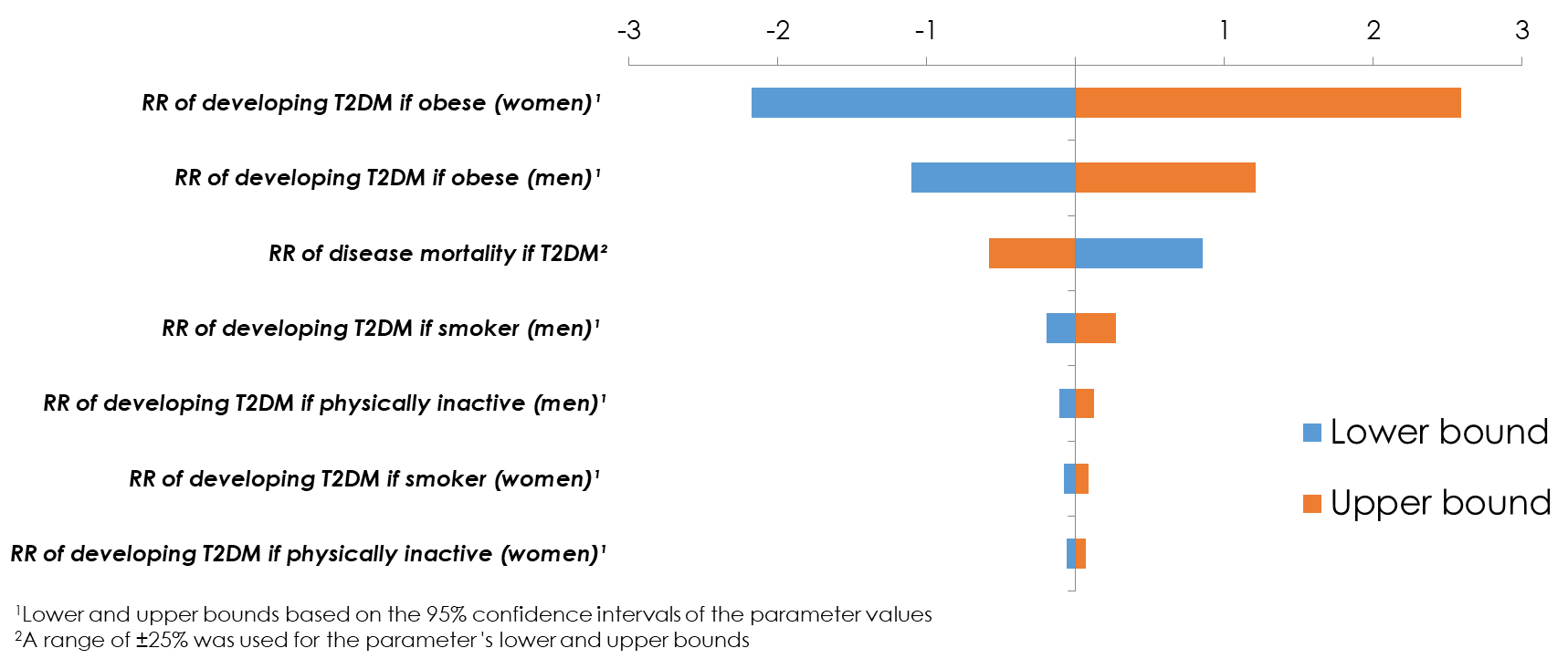
**

****Figure S8.** Projections for type 2 diabetes mellitus (T2DM) related risk factors in Jordan between 1990-2050.** The figure shows projected prevalence of A) obesity, B) smoking, and C) physical inactivity.

Figure S9. Uncertainty analyses and regularization. Uncertainty interval for the prevalence of type 2 diabetes mellitus (T2DM) in Jordan between 1990-2050 with respect to A) variations in the key structural model parameters and B) variations or errors in each data point of each survey. C) Outcome of the investigation of the potential for model overfitting by applying the regularization technique^19,20^ compared to the application of the ordinary least-square fitting method. The solid red line in panel A and B represents the mean, while the dashed lines bracket the 95% uncertainty interval.

References

1 Awad, S. F., O'Flaherty, M., Critchley, J. & Abu-Raddad, L. J. Forecasting the burden of type 2 diabetes mellitus in Qatar to 2050: A novel modeling approach. *Diabetes research and clinical practice* **137**, 100-108, doi:10.1016/j.diabres.2017.11.015 (2018).

2 The language of technical computing v. 8.5.0.197613 (R2019a). Natick, MA, USA: (The MathWorks, Inc., 2019).

3 World Health Organization. *Obesity and overweight factsheet (available at: http://www.who.int/mediacentre/factsheets/fs311/en/)*, 2015).

4 Ayoub, H. H., Chemaitelly, H. & Abu-Raddad, L. J. Characterizing the transitioning epidemiology of herpes simplex virus type 1 in the USA: model-based predictions. *BMC medicine* **17**, 57-57, doi:10.1186/s12916-019-1285-x (2019).

5 United Nations, Department of Economic and Social Affairs & Population Division. *World Population Prospects: The 2019 Revision, DVD Edition. (Available at: https://esa.un.org/unpd/wpp/Download/Standard/Population/)*, 2019).

6 World Health Organization. *STEPwise approach to noncommunicable disease risk factor surveillance in Jordan*, 2004).

7 World Health Organization. *STEPwise approach to noncommunicable disease risk factor surveillance in Jordan*, 2007).

8 Ajlouni, K., Jaddou, H. & Batieha, A. Diabetes and impaired glucose tolerance in Jordan: prevalence and associated risk factors. *J Intern Med* **244**, 317-323 (1998).

9 Ajlouni, K., Khader, Y. S., Batieha, A., Ajlouni, H. & El-Khateeb, M. An increase in prevalence of diabetes mellitus in Jordan over 10 years. *Journal of diabetes and its complications* **22**, 317-324, doi:10.1016/j.jdiacomp.2007.01.004 (2008).

10 Khader, Y. S. *et al.* Relationship between 25-hydroxyvitamin D and metabolic syndrome among Jordanian adults. *Nutr Res Pract* **5**, 132-139, doi:10.4162/nrp.2011.5.2.132 (2011).

11 Ajlouni, K. *et al.* Time trends in diabetes mellitus in Jordan between 1994 and 2017. *Diabet Med* **36**, 1176-1182, doi:10.1111/dme.13894 (2019).

12 Abdullah, A., Peeters, A., de Courten, M. & Stoelwinder, J. The magnitude of association between overweight and obesity and the risk of diabetes: a meta-analysis of prospective cohort studies. *Diabetes research and clinical practice* **89**, 309-319, doi:10.1016/j.diabres.2010.04.012 (2010).

13 Pan, A., Wang, Y., Talaei, M., Hu, F. B. & Wu, T. Relation of active, passive, and quitting smoking with incident type 2 diabetes: a systematic review and meta-analysis. *Lancet Diabetes Endocrinol* **3**, 958-967, doi:10.1016/S2213-8587(15)00316-2 (2015).

14 Fiona C. Bull, Timothy P. Armstrong, Tracy Dixon, S. H., Andrea Neiman & Pratt, M. Comparative Quantification of Health Risks. Global and Regional Burden of Disease Attribution to Selected Major Risk Factors. Chapter 10: Physical Inactivity. (available at: http://www.who.int/publications/cra/chapters/volume1/0729-0882.pdf?ua=1). (World Health Organization, 2004).

15 Nakagami, T. & Group, D. S. Hyperglycaemia and mortality from all causes and from cardiovascular disease in five populations of Asian origin. *Diabetologia* **47**, 385-394, doi:10.1007/s00125-004-1334-6 (2004).

16 International Diabetes Federation. IDF Diabetes Atlas. 3th edition. Brussels, Belgium (available at: https://www.idf.org/sites/default/files/Diabetes-Atlas-3rd-edition.pdf. Accessed on 10 Dec. 2015). (2006).

17 Zhang, P. *et al.* Global healthcare expenditure on diabetes for 2010 and 2030. *Diabetes research and clinical practice* **87**, 293-301, doi:10.1016/j.diabres.2010.01.026 (2010).

18 The World Bank. *Health expenditure per capita (current US$) (Available at: http://data.worldbank.org/indicator/SH.XPD.PCAP, Accessed April 2016)*, 2001-2013).

19 Peter Bühlmann & Sara van de Geer. *Statistics for High-Dimensional Data: Methods, Theory and Applications*. (Berlin, Heidelberg : Springer-Verlag Berlin Heidelberg, 2011).

20 Georga, E. I., Fotiadis, D. I. & Tigas, S. K. in *Personalized Predictive Modeling in Type 1 Diabetes* (eds Eleni I. Georga, Dimitrios I. Fotiadis, & Stelios K. Tigas) 37-59 (Academic Press, 2018).
